# Hydrogel-Based Micro/Nanorobots for Advanced Biomedical Applications

**DOI:** 10.3390/gels12050451

**Published:** 2026-05-20

**Authors:** Gyunhee Cho, Jongkuk Ko, Yunwoo Lee

**Affiliations:** 1 Department of Mechanical Engineering, Soongsil University, 369 Sangdo-ro, Dongjak-Gu, Seoul 06978, Republic of Korea; dung18811881@gmail.com; 2 School of Chemical, Biological and Battery Engineering, Gachon University, Seongnam-si 13120, Republic of Korea

**Keywords:** microrobot, hydrogel, biomedical applications, drug delivery

## Abstract

Micro/nanorobotics is emerging as a promising biomedical technology because of its precision, minimal invasiveness, multifunctionality, and potential to mitigate systemic adverse effects. At these ultraminiaturized scales, unique physical constraints necessitate design principles and actuation strategies distinct from those of conventional robotic systems, making material choice, structural design, propulsion mechanisms, and fabrication methods central to overall performance. In this review, we examine recent trends in micro/nanorobot development, with particular emphasis on the advantages of employing hydrogels and the current technical limitations associated with their use. Magnetic, chemical, acoustic, optical, and biohybrid propulsion strategies are comparatively analyzed, together with the material requirements and biological compatibility associated with each approach. Representative applications in drug delivery, tissue regeneration, and cancer therapy are further discussed to highlight the broad medical potential of these systems. Finally, remaining challenges related to material limitations, actuation efficiency, biocompatibility, and manufacturing scalability are identified, and future directions toward clinical translation and practical deployment are outlined. Overall, this review provides an integrated perspective on how hydrogel properties, actuation physics, fabrication strategies, and translational considerations collectively shape the development of more adaptive, biocompatible, and clinically relevant microrobotic systems.

## 1. Introduction

Continuous advances in drug development and therapeutic technologies have markedly improved the management and treatment of a broad spectrum of diseases [1,2,3,4]. Owing to progress in modern medicine, many disorders once regarded as intractable are increasingly being converted into conditions that can be managed over the long term through early diagnosis and appropriate therapeutic intervention [5,6,7]. Nevertheless, conventional pharmacotherapy still faces substantial limitations, including systemic side effects and suboptimal therapeutic efficacy [8,9,10]. These limitations arise in large part because many therapeutics are distributed nonspecifically throughout the body, resulting in insufficient accumulation at diseased sites while exposing healthy tissues to unintended toxicity. Therefore, enhancing the precision and efficiency of drug delivery has become a central objective in the development of next-generation therapeutic strategies.

In this regard, the integration of robotics into biomedical applications has opened new opportunities for site-specific and minimally invasive therapies. However, conventional macroscale robotic systems are inherently limited by their size and mechanical constraints, making them unsuitable for operation within the confined and complex environments of the human body, particularly for precise access to and manipulation within vascular networks, dense tissues, and cellular-level microenvironments [11,12]. Consequently, micro/nanorobots, which can operate at biologically relevant length scales, have emerged as promising alternatives for targeted delivery and localized therapeutic interventions [13,14].

**Figure 1:**
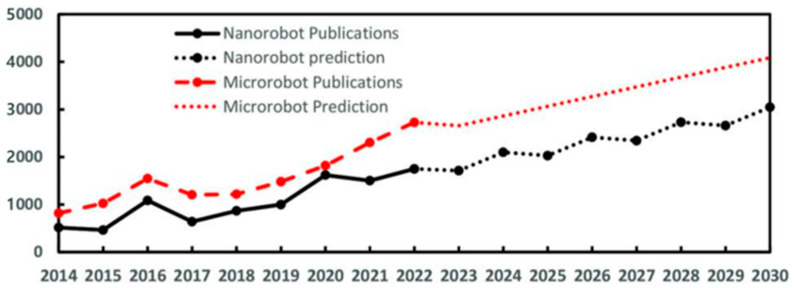
Publication trends in micro/nanorobotics based on Dimensions AI data. Annual publication counts were obtained from the analysis reported in Ref. [15], which used the Dimensions AI publication database with the search terms “Microrobot” and “Nanorobot.” The reported trend represents publication records since 2014, whereas the projected trend toward 2030 follows the extrapolation presented in the same reference. Adapted from Ref. [15] under the terms of the Creative Commons Attribution 4.0 International License.

In parallel with growing clinical demand, the control strategies of micro/nanorobots have been continuously refined. In biomedical applications, microrobots and nanorobots serve complementary roles. Microrobots offer relatively high controllability, structural complexity, and payload capacity, making them suitable for localized delivery, microgripping, cell manipulation, and minimally invasive microsurgery [14,15]. Nanorobots, by contrast, are more suitable for tissue penetration, molecular recognition, targeted drug delivery, and intracellular interventions because of their smaller dimensions, although precise tracking, propulsion, and payload loading remain challenging [16,17,18]. Thus, their biomedical use should be considered according to the required balance between controllability and tissue accessibility. Figure 1 shows that research related to micro/nanorobots has steadily increased since 2014. This upward trend suggests that research in the field of micro/nanorobots is likely to remain active in the coming years [19]. Their locomotion has evolved from two-dimensional to three-dimensional motion [20,21], from simple trajectory-following behavior to target-oriented navigation in complex environments [22], and from individual operation to collective swarm-based transport for enhanced delivery efficiency [23,24]. Owing to their ultraminiaturized dimensions, micro/nanorobots can access previously unreachable regions while minimizing damage to surrounding tissues. Their minimally invasive nature further enables faster recovery and reduced postoperative complications [11]. Beyond drug delivery, micro/nanorobots have also demonstrated strong potential in diagnostic applications, including target recognition, capture, and separation. Furthermore, micro/nanorobots have been explored in diverse therapeutic modalities, including photodynamic therapy and hyperthermia, as well as in microsurgical applications requiring manipulation at the single-cell level [12,19,25].

Despite these advances, the performance, adaptability, and clinical applicability of micro/nanorobots are strongly influenced by the choice of material platform. In particular, conventional rigid materials often limit the ability of microrobots to safely interact with soft biological tissues and to adapt to dynamic physiological environments. In this regard, hydrogels have attracted considerable attention as material platforms due to their unique combination of high water content, tissue-like mechanical properties, and tunable physicochemical characteristics [26]. Unlike rigid materials, hydrogels can undergo large and reversible deformations, enabling soft actuation and adaptive interactions with biological systems [27,28]. Moreover, their intrinsic biocompatibility, permeability, and ability to encapsulate and release therapeutic agents support their use in biomedical applications, including drug delivery, tissue engineering, and minimally invasive therapeutic systems [29]. These features position hydrogels as a key enabling material for the next generation of micro/nanorobotic platforms [30].

Compared with existing reviews that often discuss propulsion strategies, hydrogel materials, fabrication methods, and biomedical applications separately, this review presents a comprehensive, integrated, and design-oriented perspective on hydrogel-based micro/nanorobots. Specifically, we first provide an overview of the major actuation and propulsion modes of micro/nanorobots, including magnetic, chemical, acoustic, optical, and biohybrid approaches. To support mechanistic understanding, selected representative mechanisms, particularly magnetic and chemical propulsion, are further discussed with relevant theoretical descriptions that clarify the physical basis of microscale motion. This structure is intended to make the interdisciplinary field more accessible to readers from diverse backgrounds without treating propulsion as an isolated topic. We then review the principal fabrication methods employed in micro/nanorobot manufacturing and compare their defining features, advantages, and limitations. Building on this foundation, hydrogels are discussed as key material platforms for micro/nanorobots, with emphasis on their fundamental properties, advantages, current limitations, and roles in stimulus-responsive deformation, biocompatibility, biodegradability, and therapeutic functionality. Subsequently, representative biomedical applications are reviewed, particularly stimulus-responsive systems, microgrippers, and multifunctional architectures, to illustrate how hydrogel material design can be integrated with actuation and fabrication strategies. Finally, we address the remaining challenges and outline future perspectives for the clinical translation and practical implementation of hydrogel-based micro/nanorobotic technologies beyond simple locomotion-based systems.

## 2. Propulsion Mechanisms of Micro/Nanorobots

**Figure 2:**
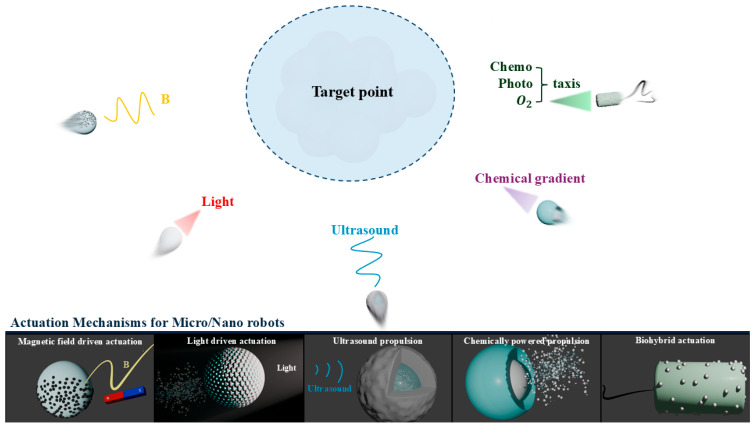
Schematic illustration of various actuation mechanisms employed in micro- and nanorobotic systems. From the upper left, the schematics depict magnetic field-driven actuation, light-driven actuation, ultrasound propulsion, chemically driven actuation, and biohybrid actuation utilizing living organisms.

To clarify the scope and organization of this review, we first introduce the representative propulsion strategies used in micro/nanorobotics, including magnetic, chemical, acoustic, optical, and biohybrid actuation. This section is not intended to provide a general survey of microrobotic propulsion alone, but rather to establish the mechanistic basis required to understand how motion, navigation, and external control are achieved at micro- and nanoscale dimensions. We then discuss hydrogels as a major material platform for biomedical micro/nanorobots, emphasizing their distinctive properties, such as high water content, softness, biocompatibility, tunable physicochemical structure, stimulus responsiveness, and capacity for therapeutic loading. Based on these two foundations, the review subsequently examines how hydrogel materials can be integrated with microrobotic propulsion and fabrication strategies to create adaptive, deformable, and functionally responsive systems. This sequential structure is intended to highlight the convergence between actuation mechanisms and hydrogel material design, thereby clarifying the unique role of hydrogels in advancing micro/nanorobots beyond simple locomotion toward biomedical functionality (Figure 2 and Table 1).

### 2.1. Magnetic Propulsion

Magnetic actuation is one of the most representative propulsion strategies for micro/nanorobots. By incorporating magnetic-responsive materials into the robotic system and applying external magnetic fields, propulsion and steering can be achieved in a remote, noncontact manner. Because biological tissues cause minimal attenuation of magnetic fields, this approach is particularly well suited for precise control in deep and confined in vivo environments [31,32]. Equations (1) and (2) [31] summarize the fundamental relationships used to describe how external magnetic fields impart force and torque to micro/nanorobots.

(1) $$\overset{⃑}{F}=\int (\overset{⃑}{M}\cdot \nabla )\overset{⃑}{B}\;dV$$

(2) $$\overset{⃑}{T}=\int \overset{⃑}{r}\times (\overset{⃑}{M}\cdot \nabla \overset{⃑}{B})\;dV+\int \overset{⃑}{M}\times \overset{⃑}{B}\;dV$$

Here, $\overset{⃑}{M}$ denotes the magnetization, representing the net magnetic dipole moment per unit volume within a material. *V* denotes the volume of the magnetic material, whereas $\overset{⃑}{B}$ represents the magnetic flux density, corresponding to the actual spatial distribution of the magnetic field [33,34]. Shape anisotropy refers to the tendency of a magnetic body to exhibit magnetically preferred directions due to its geometry, commonly termed the easy and hard axes. Because geometry-dependent demagnetizing fields make some magnetization directions energetically more favorable than others, elongated rods or ellipsoidal structures often have the long axis as the easy axis. In soft magnetic micro/nanorobots, the external magnetic field induces magnetization, and the resulting anisotropic magnetic response generates torque that tends to align the easy axis with the applied field. This geometry-dependent alignment is one of the key mechanisms underlying torque-driven actuation in soft magnetic robotic systems [31]. Building on this torque-generation mechanism, magnetic field-driven actuation of robots can be broadly categorized into magnetic force-based and magnetic torque-based modes. Force-based actuation is expressed as Equation (1) [31], indicating that a magnetic field gradient is essential, whereas torque-based actuation is described as Equation (2) [33]. In torque-driven actuation, under viscous-dominated conditions, both the hydrodynamic drag torque and the phase lag between the magnetic moment and the applied field increase with rotational speed. When the lag angle reaches 90°, the magnetic torque attains its maximum value [35]. Beyond this point, the maximum magnetic torque can no longer overcome the drag torque, resulting in a loss of synchronous rotation, referred to as step-out [36,37]. Among the various magnetic materials, iron oxide nanoparticles, particularly SPIONs (Fe_3_O_4_/γ-Fe_2_O_3_), are widely regarded as one of the most clinically translatable magnetic nanomaterials because of their favorable biocompatibility and established biomedical use. Indeed, the FDA authorization of iron oxide nanoparticle-based formulations and related clinical products supports the translational relevance of SPIONs for biomedical micro/nanorobot systems. However, their magnetization is generally lower than that of highly magnetized magnetic materials, which may necessitate additional structural or compositional optimization in applications requiring strong magnetic responsiveness [38,39,40,41,42,43,44,45].

### 2.2. Chemical Propulsion

Chemical propulsion is one of the most representative self-propulsion mechanisms in micro/nanorobots, in which propulsion is generated through interactions with the surrounding chemical environment. This actuation mode is generally categorized into two principal mechanisms: self-diffusiophoresis and self-electrophoresis [46,47]. These mechanisms are generally understood within the framework of phoretic motors, where local surface reactions perturb the surrounding concentration or potential field and generate interfacial slip flow without external actuation. Thus, chemically propelled micro/nanorobots can be viewed as systems governed by the coupling of surface reactions, solute transport, electric field generation, and low-Reynolds-number hydrodynamics [48].

#### 2.2.1. Self-Diffusiophoresis

To describe the mechanism of self-diffusiophoretic propulsion, the governing equation typically considered is the species conservation equation derived from the principle of mass conservation. This equation states that the rate of change in solute concentration at a given location is determined by the net flux entering or leaving that region. In its general form, the species conservation equation can be written as follows [49].

(3) $$\frac{\partial c_{i}}{\partial t}+\nabla \cdot j_{i}=0$$

(4) $$j_{i}=c_{i}u-D_{i}[\nabla c_{i}+\frac{c_{i}}{k_{B}T}\nabla \psi_{i}]$$

Here, $c_{i}$ denotes the concentration of species i, and $j_{i}$ represents the flux of that species. Under dilute-limit conditions, this flux is described by the generalized Nernst–Planck equation, which includes convective transport. In this expression, u denotes the fluid velocity, $D_{i}$ is the diffusion coefficient of solute species i, $k_{B}$ is the Boltzmann constant, and T is the absolute temperature. In addition, $\psi_{i}$ corresponds to the generalized interaction potential that characterizes the effective interaction between solute species i and the surrounding medium. This equation describes the solute flux, indicating that transport is governed by convection, diffusion, and migration driven by a potential gradient. By solving the species conservation equation together with the flux expression, the solute concentration distribution around the particle can be determined. Under the thin-interaction-layer approximation, the complex interfacial physicochemical interactions can be replaced by an effective slip boundary condition, leading to a surface slip velocity proportional to the tangential gradient of the relevant physical field. Accordingly, the slip velocity along the particle surface can be written as follows [50].

(5) $$u_{s}=M(I-nn)\cdot \nabla c$$

Here, M denotes the phoretic mobility, which quantifies how efficiently a local solute concentration gradient is converted into surface slip velocity [49].

#### 2.2.2. Self-Electrophoresis

The self-electrophoretic propulsion mechanism is commonly illustrated using a Pt–Au bimetallic nanorod [51]. At the Pt surface, oxidation of $H_{2}O_{2}$ releases protons into the solution and injects electrons into the metal. These electrons are transported through the nanorod toward the Au side, generating an internal current, while ionic motion in the surrounding solution establishes an ionic current, thereby closing the electrochemical circuit [52]. As a result, asymmetric ion concentration and potential distributions develop around the Pt (anode) and Au (cathode), giving rise to a self-generated electric field along the rod axis. At the Au surface, $H_{2}O_{2}$ is reduced to water through reduction reactions coupled to this electric field and ion transport, allowing the field to be sustained. The resulting electric field acts on the net charge in the electric double layer, producing an electroosmotic flow through the body force $\rho_{e}E$. Under low-Reynolds-number, force- and torque-free conditions, the nanorod propels in the direction opposite to the induced flow [53]. Current models for chemically propelled micro/nanorobots still exhibit limited predictive power, as it remains challenging to integrate multiple coupled mechanisms, including phoretic effects, bulk reactions, and confinement, without introducing excessive parameters and substantial computational complexity. In addition, the in vivo implementation of $H_{2}O_{2}$-powered systems is intrinsically constrained by concerns related to toxicity and oxidative stress, thereby motivating the development of more biocompatible propulsion strategies based on endogenous fuels, alternative catalysts, and operation under low-fuel conditions [47].

### 2.3. Light-Driven Propulsion

Light is a wireless form of energy with precise controllable directionality and readily tunable intensity, providing an effective means for the remote actuation of micro- and nanorobots. Light-driven micro/nanorobots can be rapidly controlled through light ON/OFF switching and can selectively stimulate specific locations within microscale spaces, thereby offering high spatial and temporal resolution [54,55]. Their propulsion is primarily based on light-induced reactions, in which asymmetric phenomena generated around the robot, such as concentration gradients, charge gradients, or bubble formation, give rise to the driving force [48]. Accordingly, light-responsive materials are essential for light-driven robots, with photocatalytic and photothermal materials being among the most widely used [56]. Depending on the wavelength employed, light-driven micro/nanorobots can generally be classified into ultraviolet (UV), visible light, and near-infrared (NIR) driven systems. Among these, UV light can induce strong photoreactions, which is advantageous for precise control of motion direction and for triggering aggregation or collective behaviors of multiple robots, thereby enabling cooperative functions such as collective migration, obstacle avoidance, and cargo transport. However, its relatively poor tissue penetration and potential to induce biological damage substantially limit its use in biomedical settings. By contrast, visible light offers comparatively higher biocompatibility and is therefore more suitable for biological applications. Accordingly, considerable efforts have been devoted to material and structural design strategies aimed at improving propulsion performance under visible-light irradiation [54]. NIR light is particularly attractive for biomedical applications because of its relatively high tissue penetration depth. In addition, the propulsion speed of robots can be continuously regulated by adjusting the irradiation intensity. These features make NIR-driven systems suitable for advanced therapeutic strategies, such as sequential drug release after reaching a target site or combined chemo-photothermal treatment following penetration into tumor tissues [57,58]. Overall, the speed and direction of light-driven micro/nanorobots can be flexibly controlled by modulating light intensity and irradiation direction [59]. Nevertheless, the penetration depth of light in biological tissues is inherently limited, making it increasingly difficult to sustain effective actuation in deeper tissues. Attempts to compensate for this limitation by increasing the energy input may elevate the risk of tissue damage. Consequently, light-driven micro/nanorobots still face intrinsic limitations in deep-tissue applications, which remain a major barrier to their broader translation in biomedicine.

### 2.4. Ultrasound Propulsion

Micro/nanorobots actuated by external physical stimuli without toxic chemical fuels have recently attracted increasing attention. Among these, ultrasound has attracted considerable attention as a power source owing to its safety, efficiency, tissue penetration capability, and potential for noncontact control [60]. Notably, ultrasound is already widely used in biomedical imaging, diagnosis, and physical therapy, and thus offers significant advantages as an actuation source for micro/nanorobots because of its established biocompatibility and clinical reliability. On the basis of these features, ultrasound-driven micro/nanorobots have demonstrated considerable potential in a broad range of applications, including targeted drug delivery, minimally invasive micro/nano surgery, and biosensing [61,62,63,64]. Ultrasound can generate asymmetric acoustic and flow fields around microscale structures, thereby inducing propulsion, while the resulting locomotion and control performance are strongly influenced by the robot geometry, density, material properties, and structural design. More recently, hybrid actuation strategies combining ultrasound with magnetic actuation, light-driven propulsion, or chemically catalyzed bubble recoil have also been proposed, further expanding the field in terms of enhanced functionality and precise controllability. In addition, the use of acoustic radiation forces for swarm control and collective behavior has highlighted the possibility of extending ultrasound-driven systems beyond single-robot operation toward multi-micro/nanorobot platforms [65]. Nevertheless, ultrasound-driven micro/nanorobots still face important limitations, as prolonged actuation may induce thermal effects, cavitation-related instability, and limited real-time imaging capability, thereby making precise and safe operation in vivo more challenging. Further advances in material design, acoustic field optimization, and real-time imaging and feedback control will therefore be essential to improve the safety, controllability, and clinical applicability of ultrasound-driven systems [66].

### 2.5. Biohybrid Propulsion

Biohybrid micro/nanorobots, which integrate natural microorganisms with artificial microscale structures, have attracted growing attention as a strategy to overcome the limitations of conventional artificial microrobots by combining the intrinsic self-propulsion of biological motile units with the functionality of engineered devices. The core concept of these systems is that the biological component serves as the primary source of propulsion, whereas the artificial microstructure provides directional control, propulsion enhancement, cargo loading and release, protection, and additional functionalization [67,68]. In other words, biohybrid systems exploit the motility, biocompatibility, and stimulus responsiveness of living microorganisms while achieving enhanced controllability and application versatility through engineered structures [69,70].

The operating principles of biohybrid systems can be understood from two main perspectives. First, the intrinsic motility of microorganisms or cells directly provides the driving force. For example, flagellar motion, cell membrane deformation, or cell-specific swimming mechanisms enable autonomous locomotion without the need for toxic chemical fuels. Second, the artificial microdevice can be integrated with external stimuli, such as magnetic fields, acoustic fields, or light, to enable directional guidance and expanded functionality [71]. Accordingly, biohybrid microrobots should not be viewed simply as “motile cells,” but rather as integrated actuation platforms in which biological motility is coupled with engineered control systems [72].

Representative examples include sperm-based and bacteria-based biohybrid systems. Sperm-based systems take advantage of the high motility and biocompatibility of sperm cells [73], which can not only provide propulsion but also serve as carriers for drugs or therapeutic agents. When combined with magnetic-responsive microtubes or helical microstructures, these systems can be steered by external magnetic fields, thereby enabling precise guidance toward target sites and selective cargo release. Such approaches are particularly attractive because they do not require toxic fuels and offer strong potential for biomedical applications, including assisted reproduction and targeted drug delivery [74]. Bacteria-based systems [75,76] employ highly motile microorganisms as propulsion sources and combine them with artificial microstructures to enable active locomotion within biological environments [77]. In addition to their intrinsic swimming capability, many bacteria exhibit chemotaxis, allowing them to migrate along chemical concentration gradients and thus providing the possibility of autonomous targeting. Directional control can be further introduced by incorporating magnetic nanoparticles, enabling guidance by external magnetic fields. Moreover, external stimuli such as photothermal activation may be used after therapy to regulate or eliminate bacterial activity, thereby providing an additional strategy for improving biosafety [78].

Overall, the defining feature of biohybrid systems lies in the integration of biologically derived motility with engineered control functions. This strategy can mitigate the toxicity and limited in vivo applicability associated with conventional chemically fueled micro/nanorobots, while simultaneously enabling multifunctional performance, including self-propulsion, target specificity, drug delivery, and selective release. Accordingly, biohybrid micro/nanorobots are increasingly regarded as an important future direction for precision medicine, targeted drug delivery, assisted reproductive technologies, and active therapeutic platforms capable of operating in complex biological environments [67,68,69,70,71,72,74,75,76,77,78].

## 3. Fabrication Techniques for Micro/Nanorobots

As discussed above, micro/nanorobots have emerged as next-generation microsystems capable of precise locomotion and active task execution by converting external stimuli or chemical and biological sources of energy into mechanical motion. To date, a wide range of actuation mechanisms, including magnetic, light-driven, ultrasound-based, chemical, and biological propulsion, have been proposed. However, the practical realization of these functions fundamentally depends on sophisticated fabrication processes. Depending on the required functions and intended applications, micro/nanorobots have been designed and fabricated in diverse structural forms, such as helical, tubular, needle-like, and Janus architectures. In this context, fabrication is not limited to mere shape formation, but also enables the integration of advanced functionalities, including magnetic particle incorporation, surface functionalization, porous structure formation, and biomembrane-based encapsulation. These fabrication processes ultimately determine the propulsion performance, controllability, biocompatibility, and application scope of the resulting robots. Accordingly, this section provides a systematic overview of the major fabrication methods for micro/nanorobots, together with a comparative analysis of their purposes, characteristics, advantages, and limitations. In addition, recent studies based on these fabrication strategies are reviewed to identify current research trends.

To better understand the fabrication of hydrogel-based micro/nanorobots, it is necessary to first consider the representative fabrication strategies that have been widely used in conventional micro/nanorobotics. This section therefore provides a brief overview of major fabrication approaches for micro/nanorobotic systems, including lithography-based techniques, 3D printing, self-assembly, and other microscale manufacturing methods. Rather than presenting these methods as a general fabrication survey, the discussion is intended to establish a technical foundation for understanding how such processes can be adapted to hydrogel materials. Particular attention is given to the compatibility of these fabrication strategies with the intrinsic features of hydrogels, including softness, high water content, biocompatibility, stimulus responsiveness, and the formation of crosslinked polymer networks.

**Figure 3:**
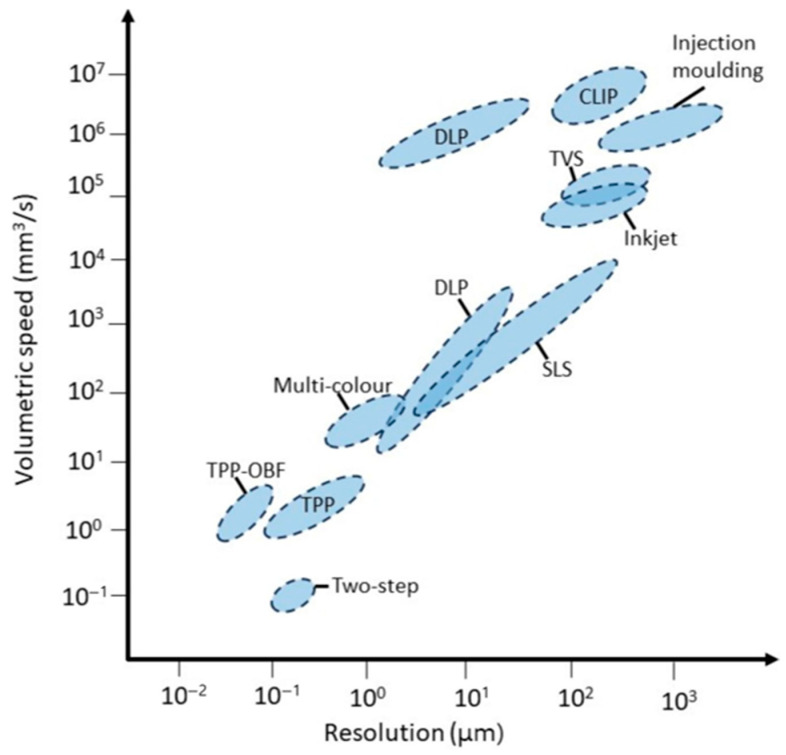
Approximate applicability ranges of 3D rapid microfabrication techniques, color-coded as a function of resolution and printing speed. Adapted from Ref. [75].

**Figure 4:**
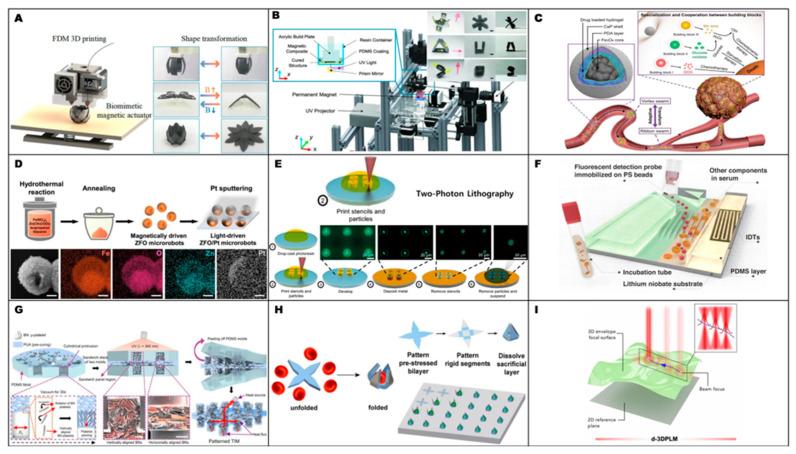
Overview of representative fabrication techniques for micro- and nanorobotic systems. (**A**) Fabrication of a bioinspired magnetic actuator using fused deposition modeling (FDM) 3D printing with a magnetic filament containing 50 wt% carbonyl iron particles (CIP). Adapted with permission from Ref. [76]. Copyright 2021 American Chemical Society. (**B**) Digital light processing (DLP)-based 3D printing of soft robots with magnetic functionality by selectively polymerizing a magnetic composite resin containing NdFeB nanoparticles within a UV-curable matrix. Adapted from Ref. [80]. (**C**) Magnetically guided self-assembly of multilayered particles composed of an Fe_3_O_4_ core, a hydrogel intermediate layer serving as a drug reservoir, and a pH-responsive shell enabling on-demand drug release during intravascular navigation under an external magnetic field. Adapted from Ref. [78]. Copyright 2021 Wiley-VCH GmbH. (**D**) Fabrication of magnetically actuated microrobots with additional optical actuation capability via sputter deposition of a Pt thin film. Adapted from Ref. [79]. (**E**) Printing of magnetically responsive particles using two-photon lithography. Adapted from Ref. [80]. © 2025 Kreienbrink et al., licensed under CC BY-NC 4.0. (**F**) On-chip separation of multiple viruses in a microfluidic channel using aptamer-functionalized microspheres, based on diffraction-based acoustofluidic surface acoustic wave (SAW) techniques. Adapted from Ref. [81]. (**G**) Fabrication of thermally conductive boron nitride (BN) micro-plates using a micromolding process. Reprinted from Ref. [82], licensed under CC BY-NC-ND 4.0. (**H**) Fabrication of a gripper actuated by differential residual stress in a bilayer structure using photolithography. Adapted from Ref. [82]. (**I**) Mold fabrication for bioresorbable devices using laser micromachining, in which the focal point of a short-pulsed laser beam precisely follows the contour of a three-dimensional shell surface. Adapted from Ref. [83].

Figure 3 presents the approximate relationship between achievable resolution and volumetric printing speed for representative 3D microfabrication techniques. Because the resolution axis is expressed in micrometers, smaller values correspond to finer feature sizes and higher resolution. As shown in the figure, techniques capable of finer feature definition are generally positioned in the lower-speed region, whereas processes operating at larger feature sizes tend to exhibit higher volumetric speeds. This relationship indicates that fabrication strategies should be evaluated not only by geometric precision, but also by throughput and scalability [79]. On this basis, the representative fabrication examples summarized in Figure 4 are discussed according to broader conceptual roles rather than as a simple sequence of individual studies. These roles include direct structural patterning for defining microrobot geometry, material and surface functionalization for introducing actuation or sensing capability, assembly-based strategies for organizing functional components, and process-oriented approaches for improving reproducibility or production efficiency. This organization helps distill a broader design principle: the selection of fabrication methods for micro/nanorobots requires balancing resolution, processing speed, material compatibility, functional integration, structural complexity, and scalability according to the intended biomedical function.

Additive manufacturing has undergone continuous technological advancement and is now recognized as a key manufacturing paradigm with the potential to fundamentally reshape conventional fabrication routes. Among the various approaches, fused deposition modeling (FDM), one of the most widely adopted and accessible 3D-printing techniques, has also been applied to the fabrication of functional robotic systems, as shown in Figure 4A. One representative study [84] demonstrated the fabrication of bioinspired actuators based on magnetically active soft materials using filament-based FDM printing. The central concept of this process lies in the preparation of magnetic filaments by blending thermoplastic elastomers with magnetic particles, followed by layer-by-layer deposition to form the desired structures in a simple and scalable manner. This work is particularly noteworthy in that structural formation, material integration, and functionalization were consolidated into a single fabrication process. The printed structures exhibited continuous shape morphing under external magnetic fields and successfully reproduced biomimetic motions such as octopus-tentacle grasping, butterfly-like flapping, and flower blooming [84]. These results indicate that FDM can serve not only as a means of geometric fabrication, but also as an effective route for the rapid production of magnetically responsive functional architectures. More broadly, this study highlights the potential of FDM 3D printing for programmable structural design and its future applicability to soft robotics and functional micro robotic systems.

As shown in Figure 4B, another notable example is a digital light processing (DLP)-based 3D-printing strategy in which magnetic nanoparticles were patterned within a UV-curable polymer matrix. This approach was proposed to address a major limitation of conventional DLP-fabricated small soft robots, which have largely relied on two-dimensional planar sheet geometries and therefore exhibit restricted deformation modes and motion capabilities. In this study, a variety of multilayered 3D architectures with overall dimensions below 10 mm were fabricated, and the volume of each segment was tailored to achieve both structural robustness and kinematic flexibility. In addition, distinct magnetization profiles were programmed into individual multilayer segments, enabling a range of motions, including gripping, rolling, swimming, and walking, through magnetic torque generated under a global magnetic field [80].

As illustrated in Figure 4C, another representative study reported a system in which externally programmed magnetic fields guided the self-assembly of individual magnetic building blocks into functional microswarms. Depending on the applied magnetic conditions, the constituent units spontaneously organized into two distinct swarm states, namely vortex-like and ribbon-like micro swarms, and these configurations could be reversibly switched within a few seconds. Such magnetically induced self-assembly and reconfiguration allowed the collective structures to adapt their morphology while navigating complex environments, including curved pathways, branched geometries, and confined spaces. Beyond simple swarm formation, the system also enabled cooperative behaviors based on task division among subpopulations with distinct functions. In particular, the self-assembled heterogeneous colloidal system achieved high delivery accessibility in structurally complex environments, while functional specialization and cooperation among the building blocks were shown to disrupt multiple growth pathways of cancer cells. This work therefore demonstrates that self-assembly can be exploited not merely as a structural formation mechanism but also as a swarm-design strategy capable of integrating collective and cooperative functionalities at the group level [85].

In another example in Figure 4D, a Janus-type ZFO/Pt microrobot was fabricated by depositing a Pt thin film onto the surface of microspheres via sputtering [86], while selectively coating only one side to introduce structural asymmetry. The asymmetric Pt layer functioned not simply as a surface coating, but as a functional interface that enabled photo-responsive self-propulsion, thereby providing the key basis for light-driven actuation. Under UV irradiation, the ZFO/Pt microrobots exhibited active motion with controllable stop-and-go behavior. Moreover, when a rotating magnetic field was applied, precise steering could be achieved in parallel with the light-driven actuation. As a result, the combination of ROS-mediated antibacterial activity and active photo motility enabled effective removal of Escherichia coli biofilms [87].

In the study depicted in Figure 4E, two-photon lithography (TPL) was employed to fabricate active particles with highly precise three-dimensional geometries, followed by the formation of arbitrary metallic patches on their surfaces [88]. TPL is particularly well suited to produce active particles with finely controlled shape and surface composition. In that work, particles were fabricated with a resolution as high as 0.2 μm, enabling the realization of electrophoretic spherical particles exhibiting tunable 3D motion, catalytic micro disks displaying chiral axial rotation, and three-dimensional magnetic particles capable of forming self-limiting microrobots through magnetic interactions. These results suggest that TPL is a powerful fabrication strategy for achieving high-resolution geometric control, functional surface design, and ultimately the production of active particles and microrobots with complex locomotion characteristics [89].

In a different line of work, a microfluidic [90] chip-based platform employing surface acoustic waves was developed to separate functional microbeads and subsequently detect cardiac troponin I via fluorescence. The microfluidic environment in Figure 4F enabled precise bead separation and target capture, while simultaneously reducing background fluorescence interference from biological fluids, thereby enhancing detection sensitivity. This study demonstrates that microfluidic techniques can serve as highly sensitive biomarker-analysis platforms by integrating target purification with signal amplification [81].

As shown in Figure 4G, another representative case involved the use of a micromolding process [91] to control the alignment of boron nitride (BN) microscale platelets dispersed within a polymer matrix, thereby producing a material with bidirectional thermal conductivity. The key feature of this process was the formation of a bifurcated orientation of BN platelets induced by the micropatterned mold geometry. The resulting alignment behavior depended on both the shape of the micropattern and the size of the BN platelets, and these parameters were further optimized through particle–fluid simulations. As a consequence, enhanced thermal conductivity was achieved in both the through-thickness and in-plane directions, while high flexibility and conformal contact with a variety of surfaces were simultaneously maintained [92].

Photolithography-based microfabrication [93] has also been used to produce untethered single-cell grippers capable of capturing individual cells. Figure 4H shows that an ${SiO/SiO}_{2}$ prestressed bilayer was employed as the hinge region, whereas thicker SiO layers were used to form rigid segments, such that differences in residual stress induced spontaneous self-folding of the structure. A major advantage of photolithography in this context is its ability to support massively parallel wafer-scale fabrication of such microscale devices. By tuning the thin-film thickness, both the folding angle and the radius of curvature could be controlled, enabling folding angles above 100° and a minimum folding radius of 765 nm. These grippers could be used either in arrayed configurations on a substrate for high-throughput cell analysis or as detached untethered structures, and their capability for capturing live fibroblasts and red blood cells was experimentally demonstrated. This study therefore shows that photolithography can function not only as a planar patterning tool, but also as a key fabrication platform for simultaneously achieving high-resolution, high-throughput manufacturing and self-folding micromechanical structures [82].

Finally, as demonstrated by Figure 4I, laser micromachining has been introduced as a fabrication route for next-generation bioelectronic interfaces. Whereas conventional UV photolithography suffers from limitations associated with hazardous chemical usage, complex multistep processing, and dependence on planar layer-by-layer structuring, this study proposed dynamically autofocused 3D pulsed laser micromachining (d-3DPLM) based on a nanosecond pulsed near-infrared laser. This method enables high-resolution ablation and patterning across diverse materials, including thin films, metal foils, and bulk metal blocks, thereby supporting the fabrication of complex three-dimensional structures and monolithic multilayer bioelectronics beyond simple planar layouts. In particular, the technique can be used to realize 3D architectures such as microneedles and deployable elements, while also enabling the formation of conformable device geometries capable of intimate interfacing with biological tissues [83].

## 4. Hydrogels as a Promising Material for Micro/Nanorobots

Hydrogels are a representative class of materials characterized by high water content, tissue-like mechanical properties, biocompatibility and tunable chemical and mechanical characteristics [94]. In particular, their swelling behavior, flexibility, stimulus responsiveness, and mass transport properties can be precisely controlled through the design of their composition and crosslinking structure, making them widely utilized as material platforms for biomedical micro/nanorobots [95]. These properties are advantageous for applications such as drug delivery, environmentally responsive behavior, soft actuation, and the realization of biocompatible interfaces. Beyond these general advantages, hydrogels uniquely bridge synthetic systems and biological environments, as their hydrated polymer networks closely mimic extracellular matrices [96]. This biomimetic nature allows seamless interaction with cells, tissues, and biofluids, which is particularly critical for in vivo microrobotic applications [97]. Therefore, this section focuses on the composition and key properties of hydrogels and examines hydrogel-based systems [98].

### 4.1. Fundamental Properties and Characteristics of Hydrogels

**Figure 5:**
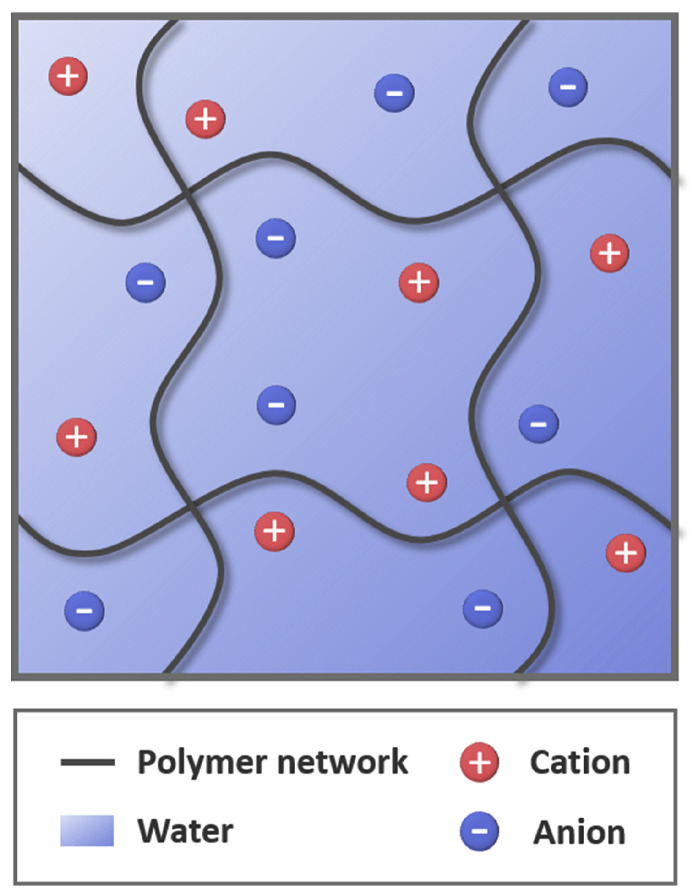
This schematic illustrates the chemical structure of a hydrogel. Hydrogels consist of a hydrophilic polymer network containing a large amount of water, and the crosslinked polymer chains form a three-dimensional elastic solid structure. In addition, the high water content enables the dissolution of solutes such as salts. Adapted from Ref. [97].

Hydrogels consist of a hydrophilic polymer network containing a large amount of water, where crosslinked polymer chains form a three-dimensional elastic structure (Figure 5). This architecture enables the retention of large volumes of water while maintaining structural integrity. The water-rich, crosslinked polymer network of hydrogels gives rise to several properties that are highly advantageous for robotic and biomedical applications [99,100].

First, hydrogels are inherently soft and flexible, and their mechanical properties can be engineered to achieve high stretchability and elasticity, allowing them to withstand large deformations without structural failure [101]. These features are particularly advantageous for hydrogel-based robots, in which energy conversion mechanisms can be embedded within the material itself, enabling sensing, environmental adaptation, shape transformation, and responsive behavior in complex environments [98,102].

Second, hydrogels exhibit excellent biocompatibility when composed of non-toxic polymers, making them suitable for applications involving direct interaction with biological tissues [103]. Their high water content provides tissue-like mechanical properties and reduces mechanical mismatch with soft biological environments, thereby minimizing damage and immune response [104].

Third, hydrogels possess intrinsic mass transport capability. The porous network structure allows the diffusion of ions, small molecules, and biomacromolecules, which is particularly advantageous for controlled drug delivery and chemical sensing applications [105]. Additionally, the dissolution of ionic species enables ionic conductivity, allowing hydrogels to function as soft conductive elements in electroactive systems. Their high water content not only imparts tissue-like mechanical properties but also allows the dissolution of salts and ionic species, thereby enabling ionic conductivity. As a result, hydrogels can be used as electrode materials in soft robots that require high stretchability [106].

Finally, hydrogels can be engineered to possess optical transparency and multifunctionality. Their low absorption of visible light allows optical signal transmission, while hybridization with nanoparticles, biomolecules, or conductive fillers further expands their functionality, enabling sensing, imaging, and actuation within a single platform [107]. Owing to these distinctive physical, chemical, and biological characteristics, hydrogels are among the most widely used materials for constructing micro/nanorobotic systems, especially in soft robotic and biomedical applications [98].

### 4.2. Actuation Mechanisms for Hydrogel-Based Microrobots

Actuation is a critical component in hydrogel-based microrobots, enabling controlled motion, deformation, and task execution in complex environments [108]. Due to their intrinsic softness and responsiveness, hydrogels are particularly well suited for stimuli-responsive and untethered actuation systems. Various actuation strategies have been developed depending on the type of external stimulus and material design. The actuation mechanisms described in Section 2 can be effectively applied to hydrogel-based microrobots, with additional advantages arising from the unique physicochemical properties of hydrogels [109]. The actuation mechanisms that are particularly compatible with hydrogel materials are presented.

#### 4.2.1. Magnetically Driven Hydrogel Microrobots

Magnetic actuation is one of the most widely utilized approaches for hydrogel-based microrobots due to its wireless controllability, deep tissue penetration, and precise spatiotemporal manipulation. By incorporating magnetic nanoparticles, such as Fe_3_O_4_, within the hydrogel matrix, microrobots can be remotely actuated through externally applied magnetic fields without requiring onboard power sources [110]. This enables a wide range of locomotion modes, including rolling, swimming, tumbling, and crawling, depending on the magnetic field configuration (e.g., rotating, oscillating, or gradient fields) and structural design of the microrobot [111]. Furthermore, magnetic actuation offers excellent controllability in complex and confined environments, which allows real-time navigation and directional control in fluidic and biological systems. This capability is particularly advantageous for biomedical applications, where precise targeting and minimally invasive operation are essential [112]. For example, magnetically actuated hydrogel microrobots have been extensively explored for targeted drug delivery, where therapeutic agents can be loaded within the hydrogel network and released at specific locations under external guidance [113]. In addition, magnetic nanoparticles embedded within hydrogels can serve multifunctional roles beyond actuation, such as providing magnetic resonance imaging (MRI) contrast [114,115], hyperthermia treatment, or responsive heating under alternating magnetic fields [116,117]. These features further enhance the versatility of magnetic hydrogel systems in theranostic applications.

#### 4.2.2. Chemically Driven Hydrogel Microrobots

pH- and chemically responsive hydrogels contain ionizable functional groups, such as carboxyl, amine, or sulfonic groups, that undergo protonation or deprotonation depending on the surrounding chemical environment [118]. These ionization processes lead to changes in charge density within the polymer network, resulting in variations in electrostatic repulsion and osmotic pressure. Consequently, the hydrogel experiences reversible swelling or deswelling, which can be translated into mechanical deformation, bending, or locomotion when appropriately structured [119,120]. The actuation behavior of these hydrogels is governed by factors such as pKa of the functional groups, ionic strength of the surrounding medium, and network crosslinking density. By designing spatial gradients in composition or crosslinking, directional and anisotropic deformation can be achieved, enabling more controlled and programmable actuation [121]. In addition, coupling chemical responsiveness with diffusion-controlled transport processes allows for time-dependent and self-regulated actuation behaviors [122]. Such chemically driven actuation mechanisms offer a useful strategy for biomedical applications, as they enable autonomous operation in response to physiological conditions without the need for external energy input. For example, pH-responsive hydrogels can exploit the acidic microenvironment of tumors or the varying pH conditions along the gastrointestinal tract to trigger site-specific drug release, structural transformation, or activation of microrobotic functions [123]. This makes them highly suitable for targeted drug delivery, smart therapeutic systems, and minimally invasive biomedical interventions [124,125]. Furthermore, chemically responsive hydrogels can be engineered to respond to specific biochemical signals, such as glucose concentration, enzymatic activity thereby enabling advanced functionalities including biosensing and feedback-controlled actuation [126,127]. These features are particularly promising for developing intelligent and adaptive microrobotic systems capable of interacting with complex biological environments.

#### 4.2.3. Electrically Driven Hydrogel Microrobots

Electric field actuation utilizes electroactive hydrogels that deform in response to an applied electric field through mechanisms such as ionic migration, electroosmosis, electrophoresis, and osmotic pressure gradients [128,129,130]. When an electric field is applied, mobile ions within the hydrogel migrate toward oppositely charged electrodes, generating internal concentration gradients and inducing asymmetric swelling or contraction. This results in macroscopic deformation, including bending, stretching, or directional motion, depending on the material composition and structural design [131]. The actuation behavior is strongly influenced by factors such as ionic conductivity, crosslinking density, solvent content, and electrode configuration. In particular, polyelectrolyte hydrogels and ionic polymer–metal composites (IPMCs) have been widely investigated due to their efficient electromechanical coupling and large deformation under relatively low voltages. By engineering anisotropic structures or layered architectures, controlled and directional actuation can be achieved, enabling complex motion patterns in microrobotic systems [130,131,132]. Electric field actuation offers several advantages, including rapid response time, precise and continuous modulation of deformation through voltage control, and compatibility with electronic interfaces [133]. These features support its use in applications such as soft actuators, artificial muscles, and responsive biomedical devices. Moreover, electric signals can be easily programmed and integrated with feedback systems, enabling real-time control and adaptive operation of microrobots [134,135]. In addition, recent studies have explored hybrid electroactive hydrogels incorporating conductive polymers, carbon nanomaterials, or metallic nanostructures to enhance electrical conductivity and mechanical performance [136,137]. Such composite systems improve actuation efficiency and enable multifunctional capabilities, including sensing and signal transduction [138].

#### 4.2.4. Light-Driven Hydrogel Microrobots

Light-driven actuation enables remote and highly precise control of hydrogel microrobots through photochemical or photothermal effects. By incorporating photoresponsive molecules (e.g., azobenzene, spiropyran) or photothermal agents (e.g., gold nanoparticles, graphene oxide, or other carbon-based materials), hydrogels can undergo reversible deformation, bending, or volumetric changes upon light irradiation [139]. These responses are typically governed by mechanisms such as photoisomerization, localized heating, or light-induced changes in hydrophilicity, which alter the internal network structure and osmotic pressure of the hydrogel [140]. Such light-induced transformations allow fast, spatially selective, and programmable actuation, enabling complex motion patterns and dynamic shape reconfiguration [141]. Depending on the wavelength and intensity of the applied light, different responses can be selectively triggered, providing a high degree of control over microrobotic behavior [55]. In particular, near-infrared (NIR) light has been widely utilized due to its relatively deeper tissue penetration and reduced photodamage compared to ultraviolet light [142]. This mechanism offers significant advantages, including high spatial resolution, rapid response time, and fully non-contact operation. As a result, light-driven hydrogel microrobots are particularly suitable for applications requiring localized and precise actuation, such as microscale manipulation, targeted drug release, optical switching, and reconfigurable soft microrobotic systems. Furthermore, light can be easily patterned and modulated, enabling parallel control of multiple microrobots or selective activation within a complex environment [143,144].

#### 4.2.5. Thermally Driven Hydrogel Microrobots

Thermal actuation relies on temperature-responsive hydrogels that exhibit reversible volume phase transitions in response to temperature changes. A representative example is poly(N-isopropylacrylamide) (PNIPAM), which undergoes a sharp and reversible transition near its lower critical solution temperature (LCST), typically around 32 °C. Below the LCST, the polymer network remains hydrated and swollen due to favorable polymer–water interactions, whereas above the LCST, hydrophobic interactions dominate, leading to rapid deswelling and network collapse [145,146]. This thermally induced change in polymer–solvent interactions results in significant volumetric variation [147], which can be harnessed to generate mechanical motion. By designing anisotropic structures or incorporating gradient crosslinking, these volume changes can be translated into directional deformation, bending, or locomotion. As a result, thermal actuation enables cyclic motion, shape transformation, and environmentally adaptive behavior in hydrogel-based microrobots [148]. Thermal actuation offers several advantages, including a relatively simple operating mechanism, ease of material design, and compatibility with a wide range of environmental conditions. It is particularly attractive for applications where environmental temperature variations can serve as a natural trigger, such as in biomedical or environmental sensing systems [149].

To overcome the limitations of single-mode actuation, recent studies have explored multi-responsive hydrogel systems that integrate multiple stimuli (e.g., magnetic + thermal, light + pH) [150,151]. These hybrid systems enable more complex and adaptive behaviors, such as sequential actuation, logic-controlled responses, and environment-specific functionality. Overall, the selection of actuation mechanism depends on the target application, required controllability, and environmental constraints.

### 4.3. Fabrication Methods of Hydrogel-Based Microrobots

The fabrication of hydrogel-based microrobots requires precise control over geometry, material composition, and functional integration. Various fabrication techniques have been developed to achieve micro/nanoscale structures with high reproducibility and functionality. The fabrication principles and methods described in Section 3 can be readily applied to hydrogel-based microrobots, with additional considerations for the unique properties of hydrogel materials. In this section, representative fabrication strategies are discussed in the context of hydrogel microrobotic systems.

Photolithographic techniques enable the fabrication of hydrogel microstructures with high spatial resolution. By selectively polymerizing photosensitive hydrogel precursors using patterned light, complex geometries can be achieved. This method is particularly useful for planar structures and high-throughput production, although it is limited in creating fully three-dimensional architectures [152,153]. Advanced additive manufacturing techniques, such as stereolithography and two-photon polymerization, allow the fabrication of complex three-dimensional hydrogel microrobots [89,154]. Laser-based methods, including two-photon polymerization and laser direct writing, allow ultra-high-resolution fabrication at the micro- and nanoscale. These techniques are capable of producing intricate structures with submicron precision, making them suitable for high-performance microrobotic systems, albeit with relatively high cost and limited throughput [89]. In particular, 4D printing, which incorporates time-dependent shape transformation, enables programmable deformation in response to external stimuli. These methods provide high design flexibility but may face challenges in scalability and material compatibility [155]. Microfluidic approaches enable the continuous and controlled fabrication of hydrogel microparticles and structures. By precisely controlling flow rates and chemical gradients, uniform and reproducible microrobots can be produced. This technique is especially advantageous for mass production and encapsulation of functional components, such as drugs or nanoparticles [156]. Self-assembly strategies utilize physical or chemical interactions to spontaneously form structured hydrogel systems [157]. These approaches are particularly attractive for miniaturized and scalable microrobot fabrication. To enhance functionality, recent approaches integrate multiple materials within a single hydrogel system [148,158,159]. Multi-material printing and sequential fabrication processes are key to realizing complex, integrated microrobotic systems.

## 5. Applications

Taken together, these clear strengths and persistent limitations suggest that the biomedical applicability of hydrogel-based micro/nanorobots is determined not only by the intrinsic functionality of the material itself, but also by how effectively its limitations can be addressed and how strategically its advantages can be exploited. On this basis, the following section focuses on representative biomedical applications in which hydrogel-based microrobots have demonstrated particular promise.

### 5.1. Hydrogel-Based Microgrippers and Stimuli-Responsive Systems

Microscale grippers are functional systems capable of precisely capturing, manipulating, and releasing small targets, showing considerable potential for biomedical applications. Their performance depends not only on structural design, but also on the selection and engineering of stimulus-responsive materials that enable controlled shape transformation in response to external stimuli or local environmental changes. Among these materials, hydrogels have emerged as particularly attractive platforms because of their responsiveness to diverse stimuli, including temperature, magnetic fields, and pH [103,160,161]. Because the actuation behavior, mechanical stability, interfacial interactions, and biocompatibility of microgrippers are strongly governed by material properties, their development should be understood as a materials-design challenge as much as a problem of structure and actuation [102]. In this regard, stimulus-responsive material-based microgrippers can perform selective physical interventions on small targets, while integrating functions such as grasping, fixation, cutting, and localized delivery within a single microsystem. These features are particularly advantageous for minimally invasive manipulation in biological environments. Accordingly, this section discusses the design principles of hydrogel-based and other stimulus-responsive, biocompatible materials, together with their biomedical applicability in microgripper systems.

**Figure 6:**
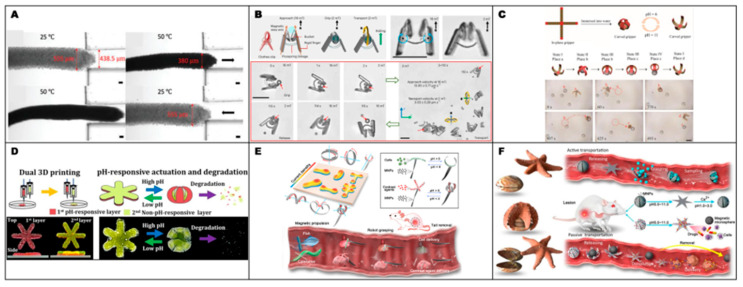
Representative micro- and nanogrippers among diverse micro- and nanorobotic architectures, highlighting their actuation mechanisms, material strategies, and functional capabilities. (**A**) A multicompartmental aquabot integrating PNIPAM-based responsive hydrogel membranes as a hydrogel–microrobot platform with stimuli-responsive behavior. Adapted from Ref. [162] (**B**) A magnetically active soft gripper that enables locomotion and material transport through environment-dependent shape morphing mediated by an active magnetic soft spring. Adapted from Ref. [163]. (**C**) A microgripper exhibiting shape morphing and enhanced magnetically driven mobility achieved by coating magnetic particles onto the surface of a stimuli-responsive hydrogel microstructure. Adapted from Ref. [164]. (**D**) A bilayer soft gripper composed of a pH-responsive alginate/gelatin/AAc hydrogel layer and a non-pH-responsive AAm supporting layer [118]. (**E**) A microgripper fabricated via a single-step anisotropic electrodeposition process, capable of simultaneously performing multiple tasks—including propulsion, grasping, and object transport-under magnetic control and ionic and pH stimuli. Adapted from Ref. [132]. (**F**) A microgripper designed to perform targeted localization, drug release, and sample collection in response to magnetic fields and environmental ionic stimuli. Adapted from Ref. [161].

Figure 6 is discussed according to the dominant stimulus governing each hydrogel-based microgripper or soft microrobotic system. The reviewed examples are therefore grouped into temperature-responsive, magnetic-field-responsive, pH-responsive, and ion-/pH-responsive platforms. Figure 6A primarily represents a temperature-responsive PNIPAM-based hydrogel microrobot with additional light and magnetic responsiveness, whereas Figure 6B illustrates a magnetic-field-responsive picospring microgripper. Figure 6C,D are classified as pH-responsive hydrogel grippers that exploit swelling/deswelling or bilayer bending for reversible opening and closing. Figure 6E,F represent ion-/pH-responsive hydrogel microrobots in which environmentally triggered shape morphing is combined with magnetic locomotion. This stimulus-based organization allows the reviewed systems to be compared through common design principles and highlights the emerging trend toward multifunctional hydrogel microrobots capable of integrating locomotion, deformation, grasping, cargo release, sensing, and environmental adaptation.

Figure 6A illustrates the use of hydrogels not merely as soft constituent materials for microrobots, but as an integrated platform that enables structural formation, stimuli responsiveness, mechanical adaptability, and electrical functionality. By combining aqueous phase separation-induced photopolymerization with all-aqueous 3D printing, the authors fabricated multicompartmental aquabots with PNIPAM-based responsive hydrogel membranes. Owing to their high-water content and porous architecture, these systems retained ultrasoftness and elasticity while exhibiting shrink-on-demand behavior in response to temperature, light, and magnetic fields. Their reversible size reduction enabled navigation through spaces substantially narrower than their original dimensions, highlighting their potential for operation in confined biological environments. In addition, integration of a conductive polymer network endowed the platform with sensing capability, suggesting that hydrogel–microrobot systems can be extended beyond actuation toward integrated sensor–actuator platforms [162].

Figure 6B presented a picospring-based soft microrobotic platform fabricated from an acrylic elastomer photoresist composed of urethane acrylate oligomer (UAO), poly(ethylene glycol) diacrylate (PEGDA), and superparamagnetic nanoparticles (MNPs). Within this material system, UAO provided a compliant elastic matrix, PEGDA served as a hydrogel component that imparted biocompatibility and affinity with aqueous environments, and MNPs enabled magnetic responsiveness. By programming nanoscale distributions of elasticity and magnetization, the authors developed highly sensitive picospring-based micromachines, including a magnetic microgripper capable of responding to forces as small as 0.5 pN. In the microgripper, a high magnetic field opened the bucket by aligning the magnetic easy axes of the fingers, whereas a reduced magnetic field allowed the arc-shaped picosprings to recover elastically and thereby close the gripper [163].

As shown in Figure 6C, Ji et al. presented a surface-coated microgripper that integrated a stimuli-responsive hydrogel with magnetic nanoparticles. In this study, the microgripper body was constructed from a pH-responsive hydrogel bilayer fabricated by two-photon direct laser writing. The upper LH layer consisted of alternating low-crosslinked (LD) and high-crosslinked (HD) hydrogel strips, whereas the lower layer was composed of a high-crosslinked hydrogel, thereby enabling shape morphing through differential swelling under pH variation. Rather than embedding Fe_3_O_4_ nanoparticles within the bulk structure, the authors coated them onto the surface, thereby taking advantage of the high surface-area-to-volume ratio at the microscale to achieve a higher magnetic material loading. As a result, the microrobot exhibited enhanced magnetically driven locomotion while preserving the intrinsic deformability of the hydrogel framework. In addition, to prevent detachment of the surface-coated Fe_3_O_4_ nanoparticles, a thin outer layer of low-crosslinked hydrogel was introduced to improve coating stability. With this materials design, the microgripper underwent reversible opening and closing in response to pH stimuli and could be remotely actuated for cargo transport under an external magnetic field. From a materials perspective, this study was notable in that the surface-coating strategy improved locomotion performance more effectively than conventional volume-embedding approaches while remaining compatible with complex three-dimensional shape-morphing designs [164].

Figure 6D illustrates a bilayer gripper fabricated by dual direct ink writing (DIW)-based 3D printing, in which a pH-responsive biodegradable hydrogel layer composed of alginate/gelatin/acrylic acid (AAc) was precisely stacked with a non-pH-responsive acrylamide (AAm)-based supporting layer. In this system, the responsive hydrogel induces self-bending and reversible opening–closing motions of the overall structure by swelling at high pH and shrinking at low pH, whereas the nonresponsive AAm layer provides mechanical support for these deformations. In particular, the swelling/deswelling behavior and responsiveness could be tuned by varying the AAc concentration and CaCl_2_ crosslinking conditions, thereby enabling the selective operation of the soft gripper under the distinct pH environments found in different organs and tissues. Moreover, on the basis of the biocompatibility and enzymatic degradability of alginate and gelatin, together with the stability of polymerized AAc, the hydrogel was presented as a biodegradable material degradable by collagenase and alginate lyase. CCK-8 and Live/Dead analyses using human dermal fibroblast (HDF) cells further indicated no pronounced cytotoxicity at the initial stage, supporting a basic level of biocompatibility [118].

Figure 6E presented a modular soft microrobot in which shape morphing and functional differentiation were simultaneously achieved by selectively loading CaCO_3_ particles and Ni magnetic nanoparticles into separate modules of an alginate-based hydrogel. Through an aniso-electrodeposition process, the authors generated a nonuniform crosslinking density within the hydrogel network and exploited the resulting swelling gradient to produce diverse three-dimensional shape-morphing behaviors, including spiraling, twisting, bending, and coiling. In this system, the sodium alginate/CaCO_3_ matrix functioned as a deformable platform responsive to pH and ionic stimuli, whereas Ni nanoparticles were selectively incorporated into a distinct propulsion module to enable magnetically driven remote actuation. In addition, cells, drugs, and fluorescent nanobeads could be selectively loaded into functional modules, thereby spatially separating propulsion capability from biological functionality [132]. Finally, environmentally responsive medical microrobots capable of adapting their structure and function to local stimuli represent another important direction.

The microrobot shown in Figure 6F was fabricated by engineering the crosslinking pattern of a hydrogel under an electric field, thereby enabling structural reconfiguration in response to magnetic actuation as well as changes in ionic concentration or pH. This adaptability allowed the robot to perform tasks such as targeting, cargo release, and sampling. In particular, the platform combined global locomotion mediated by magnetic microspheres with local opening and closing of a gripper-like structure triggered by environmental cues, demonstrating multifunctionality within a highly miniaturized format. The system was also fully biodegradable and environmentally adaptive, and its performance was validated in both in vitro and ex vivo gastrointestinal models. These findings suggest that shape-morphing microrobots may offer a promising strategy for minimally invasive medicine by enabling adaptive delivery, capture, release, and sampling in complex physiological environments [165].

### 5.2. Hydrogel-Based Micro/Nanorobotic Systems and Their Potentials

Overall, recent strategies aimed at improving the in vivo applicability of micro/nanorobots have increasingly emphasized the importance of material selection and material-interface design. Two major directions have become particularly prominent: the incorporation of biologically derived or biohybrid materials to improve immune compatibility, targeting capability, and physiological adaptability, and the adoption of biodegradable materials to ensure controlled degradation and safe clearance after task completion. Collectively, these developments indicate that the translational potential of medical micro/nanorobots depends not only on propulsion or actuation design, but also on how their material composition governs biological interactions, degradation pathways, and long-term safety in vivo. In this regard, biocompatibility- and biodegradability-oriented material design will likely remain a key basis for the clinical advancement of micro/nanorobotic systems [162].

**Figure 7:**
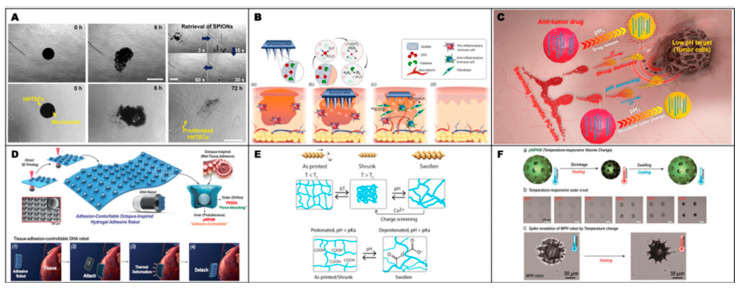
Illustrations of representative biomedical micro- and nanorobotic systems designed for targeted drug delivery, therapeutic intervention, and minimally invasive treatment. (**A**) A microrobot for stem cell delivery fabricated from biocompatible and biodegradable gelatin methacryloyl (GelMA) hydrogel. Adapted from Ref. [166]. (**B**) A biodegradable microneedle patch composed of GelMA, capable of decomposing H_2_O_2_ generated at wound sites and providing sustained oxygen release. Panels (**a**–**d**) illustrate the sequential process of wound formation (**a**), microneedle patch application (**b**), and the time-dependent degradation of the microneedles (**c**,**d**). Adapted from Ref. [167]. (**C**) PC-bots integrating pH-responsive hydrogels with magnetically driven microrobots for microenvironmental pH sensing, structural color change, and self-regulated drug delivery. Adapted from Ref. [121]. (**D**) An octopus-inspired hydrogel adhesive soft robot that combines strong wet tissue adhesion with thermally controlled detachment for minimally invasive biomedical intervention. Numbered labels (1)–(4) depict the attachment and detachment scenarios of the OHA robot on a tissue surface through controllable wet adhesion. Reprinted from Ref. [142], licensed under CC BY-NC 4.0. (**E**) Hydrogel-based magnetic microscrews and microrollers enable multistimuli-responsive size transformation, confined-space adaptation, and controlled capillary occlusion while maintaining magnetic actuation. Adapted from Ref. [146]. (**F**) A hydrogel-based pollen grain-inspired microrobot enables magnetically guided locomotion, thermally controlled anchoring, and pH-responsive cargo release through decoupled multifunctional design. Adapted from Ref. [147].

As described above, for the practical in vivo application of micro/nanorobots, it is essential not only to improve actuation performance and functional precision, but also to ensure stable adaptation and safety in complex biological environments. From this perspective, hydrogels are regarded as promising material platforms because their relatively mild physicochemical characteristics and biocompatible nature can help reduce incompatibility with biological environments. In addition, when their surface and internal structures are appropriately designed, hydrogels may help mitigate nonspecific immune responses and improve functional persistence [163,164]. Hydrogels can also be designed on the basis of biodegradable polymers, which is advantageous for reducing unexpected toxicity or chronic adverse effects caused by post-administration retention in the body and for enhancing subsequent clearance [165]. Accordingly, hydrogel-based micro/nanorobots are considered a promising material approach that can address both biocompatibility and biodegradability. Figure 7 illustrates representative microrobots fabricated from biocompatible and biodegradable hydrogels.

To improve clarity and guide the reader through the following discussion, the hydrogel-based microrobotic systems in Figure 7 are first classified according to their dominant biomedical material characteristics. Figure 7A,B are categorized as systems that integrate both biocompatibility and biodegradability, whereas Figure 7E is mainly associated with biodegradability. In contrast, Figure 7C,D,F are grouped as biocompatibility-oriented systems, as they emphasize biologically compatible sensing, tissue interaction, adhesion, or therapeutic operation. This classification is intended to help readers understand the main material design focus of each system before the individual examples are discussed in detail.

In parallel with biocompatibility, biodegradability has emerged as a critical design requirement for the safe clinical translation of micro robotic systems. As shown in Figure 7A, one representative example is the development of spherical gelatin methacrylate (GelMA) microrobots that can be mass-produced in microfluidic channels and subsequently degraded enzymatically. These fully biodegradable microrobots were loaded with human nasal turbinate stem cells (hNTSCs) and exhibited precise rolling motion under a rotating magnetic field. Following collagenase-mediated degradation, the encapsulated stem cells were released, proliferated, and differentiated into neuronal cells, while electrophysiological activity measured by multielectrode arrays supported their potential as a cell-therapy delivery platform. Such systems demonstrate how biodegradability can be integrated with controlled actuation and regenerative function [166].

Biodegradable microneedle arrays (MNAs) based on gelatin methacryloyl, as illustrated in Figure 7B, were designed to combine sufficient mechanical robustness for tissue insertion with gradual biodegradation after administration. Upon exposure to interstitial fluid and wound exudate, the system released oxygen in a controlled manner, thereby supporting tissue repair without requiring device retrieval. Through compositional optimization, both oxygen-release behavior and biocompatibility were improved, and in vivo studies confirmed that the optimized oxygen-generating MNAs did not induce tissue damage or impair healing. Transcriptomic analysis further revealed activation of beneficial wound-healing pathways. Taken together, these findings suggest that biodegradable oxygen-releasing MNAs constitute a promising platform for chronic wound therapy, as they combine therapeutic functionality with safe post-implantation degradation [167].

Figure 7C presents an intelligent platform that integrates a pH-responsive hydrogel with magnetically driven microrobots, highlighting the significance of employing the hydrogel not merely as a soft constituent material, but as a functional body responsible for environmental sensing, structural color modulation, drug loading and release, and basic biocompatibility. The authors fabricated magnetic photonic-crystal microrobots (PC-bots) by incorporating one-dimensional periodic assemblies of Fe_3_O_4_ nanoparticles into poly(AA-co-AM)-based hydrogel microspheres. Through this design, the robots were able not only to achieve precise motion and swarm self-organization under external rotating magnetic fields, but also to perform real-time visual pH sensing via hydrogel swelling and deswelling, which altered the interparticle spacing and lattice constant and thereby changed the structural color. In addition, by exploiting the electrostatic interactions between the carboxyl groups of the hydrogel scaffold and DOX, the system enabled high-capacity drug loading and faster release in acidic tumor microenvironments, thus integrating active swarming locomotion, microenvironmental sensing, and self-regulated drug delivery within a single microrobotic platform. Notably, the authors further showed that bare PC-bots exhibited negligible cytotoxicity toward MCF-7 cells over a range of concentrations, and maintained a hemolysis rate below 1% even at high concentrations, suggesting that the hydrogel-based outer layer contributes to a basic level of biocompatibility [121]. Recent efforts toward minimally invasive intervention using wireless small-scale medical robots have highlighted the importance of achieving both strong tissue adhesion and controllable detachment in biological environments.

As displayed in Figure 7D, these functions were realized through a hydrogel-centered materials design by developing a small-scale soft robot equipped with an octopus-inspired hydrogel adhesive (OHA). Rather than serving merely as a passive structural component, the hydrogel was engineered as the key functional material governing interfacial adhesion and release behavior. Specifically, hydrogels with different Young’s moduli were combined to prevent collapse of the octopus-inspired microstructures during preloading, thereby enabling strong and biocompatible wet adhesion on biological tissues. In addition, a polyethylene glycol diacrylate (PEGDA) hydrogel was used to form the sucker wall, while a poly(N-isopropylacrylamide) hydrogel was introduced as dome-like protuberances within the wall. This thermoresponsive PNIPAM component underwent temperature-dependent volume changes, allowing robust tissue attachment under wet or underwater conditions while also enabling facile and controlled detachment by thermal stimulation. As a result, the robot achieved repeatable attachment and release on biological tissues and effectively performed biomedical functions in vivo. Collectively, this study demonstrates that hydrogel design can directly determine not only the mechanical compliance and biocompatibility of small-scale soft robots, but also their wet-adhesive performance and stimulus-responsive detachment behavior [142].

Figure 7E schematically shows that the hydrogel is not merely used as a soft structural matrix, but functions as the central material platform that enables reversible or programmed size transformation in response to temperature, pH, and divalent cations, while maintaining magnetic controllability. On this basis, the hydrogel microscrews exhibited reversible swelling and shrinking over multiple cycles, whereas one-way size-controllable variants showed large unidirectional expansion of up to 65% of their initial length, followed by enzymatic degradability for removal. At the same time, the magnetically responsive microrollers demonstrated spatial adaptability by traversing narrow channels and showed the potential for controlled occlusion of small capillaries. These results highlight that hydrogel integration can substantially expand the functional repertoire of magnetic micromachines by coupling mobile magnetic control with stimulus-responsive reconfiguration, thereby enabling biomedical tasks such as targeted obstructive intervention and lab- or organ-on-a-chip manipulation [146].

Figure 7F presents a hydrogel-based multifunctional microrobot in which distinct hydrogel components were rationally integrated to decouple locomotion, on-demand anchoring, and cargo release within a single microsystem. Specifically, the robot consisted of a FePt nanoparticle-embedded PETA spike structure for magnetic actuation, a pNIPAM outer shell for temperature-responsive attachment control, and a pNIPAM-AAc inner sphere for pH-responsive cargo release. The pNIPAM outer shell shrank by up to 49% upon heating, thereby exposing the spike structures and allowing controllable attachment to biological surfaces, whereas the pNIPAM-AAc inner carrier underwent pH-dependent swelling of up to 300%, enabling on-demand active cargo release. At the same time, the FePt-loaded magnetic structure provided torque-driven rolling locomotion and steering under rotating magnetic fields, with a reported maximum translational speed of 532 µm s^−1^. Collectively, this hydrogel-based architecture demonstrates how multiple responsive hydrogel elements can be spatially integrated to achieve decoupled multifunctionality in biomedical microrobots [147].

### 5.3. Current Challenges and Limitations of Hydrogel Systems

Although hydrogels offer notable advantages such as excellent biocompatibility, stimulus responsiveness, and flexibility, several limitations still need to be addressed before they can be broadly applied in practical soft robotic and biomedical systems. First, because hydrogels are water-rich materials, water evaporation readily occurs under ambient conditions, which can alter their swelling state and mechanical behavior depending on the surrounding humidity [168]. While such environmental sensitivity may be useful in certain sensing applications, it becomes undesirable in systems that require precise actuation and stable performance [169]. In addition, nonuniform crosslink density distribution can reduce optical transparency and mechanical strength, thereby limiting the consistency of material performance [170]. Although the high water content of hydrogels provides tissue-like properties, it can also lead to low mechanical strength, poor adhesion, and insufficient structural stability, which impose constraints on practical robot design.

In addition to initial mechanical stability, fatigue behavior and long-term durability are important considerations for hydrogel-based microrobots that undergo repeated actuation cycles. Repeated swelling/deswelling, bending, gripping, or locomotion-associated deformation may lead to hysteresis, accumulation of network damage, crack initiation or growth, and gradual degradation of actuation performance [166]. In multifunctional hydrogel systems, cyclic deformation may also affect the interfacial stability between the hydrogel matrix and embedded functional components, such as magnetic particles, conductive networks, or drug-loaded compartments. Therefore, future hydrogel microrobot designs should evaluate not only short-term stimulus responsiveness, but also cyclic actuation stability, fatigue resistance, and long-term retention of structural and functional performance under physiologically relevant conditions [102,167].

From a manufacturing perspective, hydrogels face challenges in achieving highly precise fabrication of complex structures and in integrating multiple materials. In the past, slow multistep processes based on free-radical polymerization also made it difficult to precisely control monomer composition. Although improved methods such as one-pot synthesis have recently been introduced, advanced 3D printing techniques and further process optimization are still required [171].

Furthermore, the development of electroactive hydrogel systems requires electrodes that simultaneously provide high conductivity and high stretchability, yet a universally applicable solution has not been fully established. Difficulties in sterilization and the high cost of processing also remain important barriers to biomedical use. Therefore, for hydrogel-based systems to evolve into more practical and reliable soft robotic platforms, continued improvements are needed in material composition, water stabilization, fabrication precision, electrode integration, and long-term reliability [91,170].

## 6. Conclusions

Recent advances in micro/nanorobotics indicate that the field is evolving from simple demonstrations of locomotion toward biologically relevant systems that integrate actuation, materials, fabrication, and therapeutic function. As reflected in this review, magnetic, chemical, acoustic, optical, and biohybrid propulsion modes each provide distinct advantages, yet their practical value is ultimately determined by how effectively they are combined with appropriate material platforms and biomedical requirements. In this regard, the present analysis emphasizes that micro/nanorobot design should be understood as a coupled problem of actuation physics, structural engineering, and translational compatibility rather than as a question of propulsion alone.

Within this broader framework, hydrogels stand out as one of the most important material platforms in micro/nanorobotics because their water-rich, crosslinked polymer networks offer softness, flexibility, biocompatibility, transparency, ionic conductivity, and tunable responsiveness to external stimuli. These features make hydrogel-based systems particularly attractive for biomedical tasks requiring controlled deformation, adaptive interfacing with tissues, and integrated therapeutic functions. At the same time, the literature reviewed here shows that hydrogel-based micro/nanorobots still face important limitations, including weak mechanical robustness, dehydration-related instability, fabrication complexity, and the difficulty of integrating conductive yet stretchable functional elements. Thus, the practical significance of hydrogels lies not only in their intrinsic advantages, but also in the possibility of refining their structure, composition, and processing to better balance responsiveness with stability and manufacturability.

A further outcome of this review is that the medical scope of micro/nanorobotics is expanding beyond mobile delivery systems toward multifunctional platforms capable of active and localized intervention. In particular, the inclusion of gripper-type microrobotic systems highlights how material choice directly governs not only motion, but also grasping, fixation, release, and microscale manipulation in tissue- and cell-level settings. Table 2 summarizes hydrogel-based soft actuator systems according to stimulus type, actuation mode, response characteristics, and application domains, illustrating how material design can be linked to functional actuation and biomedical use.

A comparative evaluation of the systems reviewed in this article further shows that no single propulsion or material strategy is universally optimal. Magnetic systems offer precise wireless control and relatively favorable tissue penetration, but they require external field-generation equipment and careful consideration of magnetic particle loading, long-term retention, and imaging compatibility. Chemically driven systems can provide autonomous motion and high local thrust, yet their biomedical translation is often limited by fuel dependence, reaction by-products, and difficulties in controlling motion under physiological conditions. Acoustic and optical actuation provide non-contact spatiotemporal control, but acoustic systems require careful management of cavitation, heating, and spatial resolution, whereas optical systems are constrained by limited tissue penetration and potential photothermal damage. Biohybrid systems benefit from intrinsic motility, biological sensing, and tissue compatibility, but their reproducibility, immune response, storage stability, and regulatory control remain challenging. Furthermore, hydrogel-based micro/nanorobots provide distinctive advantages in softness, water-rich biocompatibility, stimulus responsiveness, and tissue-like mechanical properties; however, their relatively limited mechanical strength, dehydration instability, and difficulty in integrating conductive, magnetic, or therapeutic components without compromising flexibility remain major limitations.

Therefore, the future of hydrogel-based micro/nanorobotics should move beyond the pursuit of locomotion alone and focus on balancing actuation efficiency, material robustness, biological safety, fabrication scalability, and therapeutic function. Future studies should prioritize mechanically reinforced yet biodegradable hydrogel networks, anti-dehydration and immune-evasive surface designs, and hybrid architectures that integrate magnetic, acoustic, optical, or electroactive components without compromising softness and biocompatibility. In parallel, standardized evaluation criteria and real-time imaging or feedback control strategies will be essential for advancing hydrogel micro/nanorobots from proof-of-concept demonstrations toward clinically relevant therapeutic platforms.

From a translational perspective, future hydrogel-based micro/nanorobots should be evaluated not only in terms of actuation and therapeutic performance, but also with respect to biosafety, biodegradation, regulatory requirements, and scalable manufacturing. Biosafety assessment should include immune responses, hemocompatibility, inflammatory reactions, long-term retention, biodistribution, clearance, and the potential toxicity of incorporated functional components. Biodegradation profiles should be predictable and matched to the intended therapeutic window, with degradation products that are non-toxic and safely cleared from the body. In parallel, regulatory translation will require standardized characterization of material composition, sterilization compatibility, batch-to-batch reproducibility, actuation safety, and in vivo fate. Therefore, scalable, reproducible, and quality-controlled manufacturing strategies will be essential for advancing these systems from laboratory-scale demonstrations toward clinically applicable biomedical platforms.

Taken together, the studies discussed in this review support the conclusion that hydrogel-centered material design, when rationally integrated with suitable actuation mechanisms and fabrication strategies, offers a meaningful path toward more adaptive, biocompatible, and clinically relevant micro/nanorobotic systems. In particular, the development of hydrogel formulations that simultaneously address mechanical robustness, stimulus-responsive actuation, and immune-evasive surface design will be critical for bridging the gap between laboratory proof-of-concept and clinical deployment.

## Figures and Tables

**Table 1:** Comparison of representative actuation methods for micro/nanorobots, including their major advantages and limitations. Reproduced from Ref. [15] under the terms of the Creative Commons Attribution 4.0 International License. No changes were made.

Actuation Method	Advantages	Disadvantages
Magnetic field	Powerful penetrating power, no harm to living biological systems, fast actuation speed	Unavoidable safety problems when applying high intensity magnetic fields over 8 T
Electric field	Adjustable electric field intensity, powerful penetrating power	Imitations on biomedical applications of electrodes
Light field	Precise targeting, various controlling parameters, such as power density, wavelength, pulse frequency, polarities and so on	Irreversible harm to biological materials, poor light transmission
Acoustic field	Adjustable for different heights, biocompatible within a certain frequency range, powerful penetrating power, and actuation force	Prolonged actuation time may cause thermal effects, low imaging, and cavitation effect
Chemical	Larger and faster spread	Lack of feedback fuel safety issues, nontoxic urea, biocompatibility
Biological	Good biocompatibility, cause little rejection reaction	Need to maintain activity, limited range, need ultra-clean operating environment

**Table 2:** Representative alginate-based hydrogel soft actuators classified by stimulus type, actuation mode, response characteristics, and application domains. Adapted from Ref. [168].

System/Material	Stimulus	Max Actuation Strain (%)	Blocking Force (N)	Response Time	Actuation Mode	Application Domain	Reversibility/Cyclic Use	Reference
P(NIPAm-co-NDEAm)	Temperature, pH, light	~50%	Not reported	1–3 min	Volumetric swelling/deswelling	Controlled agrochemical release	High, multi-cycle-tested	[169]
Magnetic alginate beads	Magnetic field	~10%	Not reported	Instantaneous	Magnetic rotation/translation	Targeted drug delivery	High, magnetic field-controlled	[91,170,171]
Braided hydrogel muscle	Temperature (cooling from 60 °C)	7–8%	5–6 N	Slow (minutes)	Contraction due to swelling	Artificial muscles/soft robotics	Moderate (fatigue observed)	[172]
Electroactive polycarbazole	Electric field (low voltage)	1–2%	Low (µN-mN)	Seconds	Voltage-induced deformation	Electroactive sensing or actuation	Limited (electrochemical fatigue)	[173]
Photothermal GO–based bilayers	NIR light	15–20%	Not reported	Fast (~seconds)	Bending/folding due to heating	Microrobotics, biomedical folding	Good, NIR-cycled	[174]
Magnetic micromotors	Magnetic field	~12%	Not reported	Sub-second rotation	Magnetic propulsion	Remote-controlled microswimmers	Yes, in fluid environment	[175,176]
pH-responsive Ca–based beads	pH variation (acidic)	5–10%	Not applicable	2–5 min	Swelling and gel softening	Environmental remediation	Yes, but pH-limited	[177,178]
Triboluminescent EuD4TEA–based beads	Mechanical impact	Swelling-dependent	Not applicable	Instantaneous flash	Optical emission due to deformation	Mechanical sensing and diagnostics	No, single flash	[179]
Piezoelectric microspheres	Electric field (piezoelectric)	0.5–1%	Very low	Milliseconds	Electric field-induced deformation	Biosignal-responsive systems	Yes, piezoelectric loop	[180]

## Data Availability

No new data were created or analyzed in this study. Data sharing is not applicable to this article.
